# UPF1, a Conserved Nonsense-Mediated mRNA Decay Factor, Regulates Cyst Wall Protein Transcripts in *Giardia lamblia*

**DOI:** 10.1371/journal.pone.0003609

**Published:** 2008-10-31

**Authors:** Yi-Hsiu Chen, Li-Hsin Su, Yu-Chang Huang, Yi-Ting Wang, Yu-Yun Kao, Chin-Hung Sun

**Affiliations:** Department of Parasitology, College of Medicine, National Taiwan University, Taipei, Taiwan, Republic of China; Wellcome Trust Sanger Institute, United Kingdom

## Abstract

The *Giardia lamblia* cyst wall is required for survival outside the host and infection. Three *cyst wall protein* (*cwp*) genes identified to date are highly up-regulated during encystation. However, little is known of the molecular mechanisms governing their gene regulation. Messenger RNAs containing premature stop codons are rapidly degraded by a nonsense-mediated mRNA decay (NMD) system to avoid production of non-functional proteins. In addition to RNA surveillance, NMD also regulates thousands of naturally occurring transcripts through a variety of mechanisms. It is interesting to know the NMD pathway in the primitive eukaryotes. Previously, we have found that the giardial homologue of a conserved NMD factor, UPF1, may be functionally conserved and involved in NMD and in preventing nonsense suppression. In this study, we tested the hypothesis that NMD factors can regulate some naturally occurring transcripts in *G. lamblia*. We found that overexpression of UPF1 resulted in a significant decrease of the levels of CWP1 and cyst formation and of the endogenous *cwp1-3*, and *myb2* mRNA levels and stability. This indicates that NMD could contribute to the regulation of the *cwp1-3* and *myb2* transcripts, which are key to *G. lamblia* differentiation into cyst. Interestingly, we also found that UPF1 may be involved in regulation of eight other endogenous genes, including up-regulation of the *translation elongation factor* gene, whose product increases translation which is required for NMD. Our results indicate that NMD factor could contribute to the regulation of not only nonsense containing mRNAs, but also mRNAs of the key encystation-induced genes and other endogenous genes in the early-diverging eukaryote, *G. lamblia*.

## Introduction

*Giardia lamblia* is a major cause of outbreaks of waterborne diarrheal disease worldwide, which contributes greatly to malnutrition and malabsorption in children [1]–[3]. Like *Entamoeba histolytica* and other intestinal protozoan parasites, *G. lamblia* undergoes differentiation from a pathogenic trophozoite form into a resistant infectious cyst form [3], [4]. Cyst can survive in the hostile environment and infect a new host because they have a resistant extracellular wall.

Because of the importance of the cyst wall, a lot of researches are focusing on identifying and understanding its key components, cyst wall proteins (CWPs) [5]–[7]. Interestingly, three *cwp* genes identified to date are highly up-regulated during encystation [5]–[7]. There is little understanding of the molecular mechanisms governing their transcriptional regulation. A Myb family transcription factor (Myb2) is encystation-induced and is involved in coordinating upregulation of the *cwp1-3* genes [8]. Two GARP family transcription factors may be involved in transcriptional regulation of many different genes including the encystation-induced *cwp1* gene and constitutive *ran* gene [9]. An ARID family transcription factor can bind to specific AT-rich Inr sequences and function as an important transactivator in the regulation of the *cwp1* gene [10]. However, little is known about regulation of mRNA stability of the *cwp* genes during giardial growth and differentiation.

In late-branching eukaryotes, either a frameshift or a nonsense mutation often leads to rapid degradation of the gene's mRNA by a nonsense-mediated mRNA decay (NMD) pathway [11]–[13]. This surveillance system protects cells from the production of non-functional proteins by eliminating mutant mRNAs. The NMD pathway is present in yeast, plants, *Caenorhabditis elegans* and mammals [11]–[13]. NMD factors such as up-frameshift 1 (UPF1), UPF2 and UPF3 have been identified in yeast, *C. elegans*, mice and humans [14]–[19]. They have been shown to interact together and form a complex [20], [21]. Mutations in *upf* genes stabilize mRNAs with nonsense mutations [22], [23]. UPF1 is one of the most conserved NMD factors [11]–[13]. NMD is a translation-dependent event since its mechanism depends on the recognition of the nonsense mutations by the translational machinery [13]. Studies have shown that NMD factors including UPF1 enhance translation termination at a nonsense codon through interaction with the termination release factors [20], [24]–[26].

In addition to RNA surveillance, NMD factors also function in regulating the abundance of some naturally occurring mRNAs [22], [27]. Hundreds or thousands of wild-type NMD targets have been identified in yeast and humans [28], [29]. Most of them are up-regulated, some of them are down-regulated in *upf* null mutant [29].

*G. lamblia* is of biological interest in understanding the progress of eukaryotic evolution [30]–[33]. It has fewer cellular components for DNA synthesis, transcription and RNA processing, possibly due to their divergence or their functional redundancy with other proteins in some pathways [33]. Blast searches of the *G. lamblia* genome databases identified matches for UPF1, which is the most conserved NMD factor but no matches for UPF2 and UPF3 and some other NMD factors [34]. Interestingly, our previous results showed that: i) the levels of the nonsense transcripts were lower in *G. lamblia*; ii) the aminoglycoside G418 had an inhibiting effect on NMD in *G. lamblia*, similar to the effect of aminoglycosides on inhibiting NMD in late-branching eukaryotes; iii) Giardial UPF1 functioned in reducing the levels of nonsense-containing transcripts and in enhancing fidelity of translation termination [34]. Therefore, the NMD phenomenon could be present in *G. lamblia*. However, *G. lamblia* does not have some of the components of the NMD pathway and the reduction levels of the nonsense transcripts observed in *G. lamblia* are lower than those in late-branching eukaryotes, suggesting that the NMD system in *G. lamblia* may be less-functional.

Previously, we have found that the expression of the *upf1* gene was induced in cells expressing the *luciferase* gene with a nonsense mutation [34]. In this study, we found that the nonsense mutation triggered a decrease in *cwp1* and *cwp2* mRNA levels and there was a reverse correlation between the expression levels of the *cwp1/2* and *upf1* mRNA. We also found that overexpression of UPF1 reduced the levels of CWP1 and cyst formation and reduced the mRNA levels and stability of the *cwp1*, *cwp2*, *cwp3*, and *myb2* genes. In addition, we found that the expression of other five genes was increased and that of other three genes was decreased by the UPF1 overexpression. For example, the *translation elongation factor* mRNA was increased by the UPF1 overexpression. This could be a new example of an NMD target whose product increases translation which is required for NMD. Our findings provide new insights into regulation of the giardial cyst wall protein genes and other endogenous genes.

## Results

### NMD can be monitored by a constitutive promoter system

**Figure 1:**
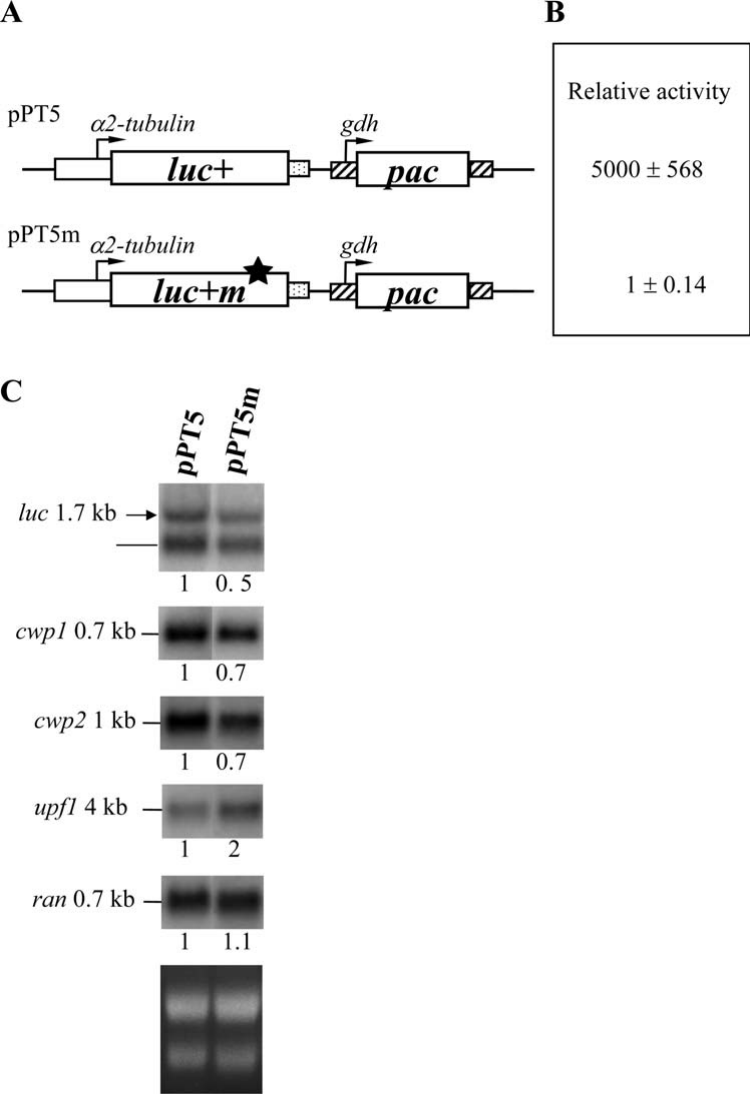
Down-regulation of the *cwp* genes in cells expressing the *luciferase* gene with a nonsense mutation. (A) Diagrams of the pPT5 and pPT5m plasmids. The *pac* gene (open box) is under the control of the 5′- and 3′-flanking regions of the *gdh* gene (striated box). The luciferase reporter gene (*luc+*, open box) is under the control of the 5′-flanking region of the *α2-tubulin* (open box) and the 3′-flanking region of the *ran* gene (dotted box), respectively. The arrows show the directions of gene transcription. Plasmids pPT5 and pPT5m encode a wild-type *luciferase* gene (*luc+*) with 550 amino acids and a luciferase mutant (*luc+m*) in which Leu 411 was mutated to a termination codon (marked by a star) and Asp 500 was mutated to Asn, respectively. (B) Nonsense mutation leads to a decrease of luciferase activity. After stable transfection with the pPT5 and pPT5m constructs, luciferase activity was measured in vegetative cells. The activity of pPT5 transfectants relative to pPT5m transfectants is presented. Values are shown as mean±standard error. (C) Down-regulation of the *luciferase* gene with a nonsense mutation. Total RNA blots made from vegetative transfectants were hybridized with specific gene probes as indicated (upper panels). Ribosomal RNA loading controls are in the bottom panel. Representative results are shown. Two different *luciferase* transcripts, a 1.7 kb full-length transcript (shown by an arrow) and a 1.2 kb transcript (shown by a line), were detected in the pPT5 and pPT5m transfectants. The numbers show the relative activity, which reflects expression relative to that in controls.

NMD is related to the presence of a premature stop codon or not [11]–[13]. We have tested NMD effect using a *luciferase* gene with or without a premature stop codon under the control of the encystation-induced *cwp1* promoter [34]. We found that NMD could be present in *G. lamblia* because the results showed that the mRNA produced from the *luciferase* gene with a stop codon under the control of the *cwp1* promoter was decayed compared with wild type luciferase mRNA [34]. We further used a constitutive promoter to test the NMD. We prepared puromycin-based constructs pPT5 and pPT5m in which the wild type *luciferase* gene (*luc+*) and the *luciferase* gene with a stop codon (*luc+m*) are controlled by the *α2-tubulin* promoter, respectively (Fig. 1A), and stably transfected them into *G. lamblia*. The level of luc+m activity in the pPT5m cell line was reduced by approximately 5,000-fold, relative to that of the wild-type luc+ in the pPT5 cell line (Fig. 1B), indicating that luc+m could be non-functional. This result is similar to the data we previously reported for the luc+m under the control of the *cwp1* promoter [34]. The level of luciferase mRNA was lower by ∼50% (*P*<0.05) in the pPT5m transfectants compared with that in the pPT5 transfectants (Fig. 1C, lanes 1 and 2), indicating that the presence of a nonsense mutation in *luc+m* triggered a decrease in mRNA levels (NMD). Therefore, NMD can be monitored by a constitutive promoter system. The results from the *α2-tubulin* and the *cwp1* promoters [34] similarly indicate that NMD could be present in *G. lamblia*. As a control, the *ran* mRNA levels did not change in the pPT5m transfectants compared with the pPT5 transfectants (Fig. 1C, lanes 1 and 2).

### Reverse correlation between the expression level of the *cwp1/2* and *upf1* mRNA

Previously we found that the nonsense mutation triggered an increase in *upf1* mRNA levels [34]. In this study, we also found that the levels of the *upf1* mRNA increased by ∼2-fold (*P*<0.05) in the pPT5m cell line compared with the pPT5 cell line (Fig. 1C). Interestingly, the levels of the *cwp1* and *cwp2* mRNA were lower by ∼30% (*P*<0.05) in the pPT5m cell line compared with the pPT5 cell line (Fig. 1C). Similarly decreased *cwp1* or *cwp2* mRNA levels were also detected in the pPW1m cell line that expressed the *luciferase* gene with a premature stop codon under the control of the *cwp1* promoter (data not shown) [34].

**Figure 2:**
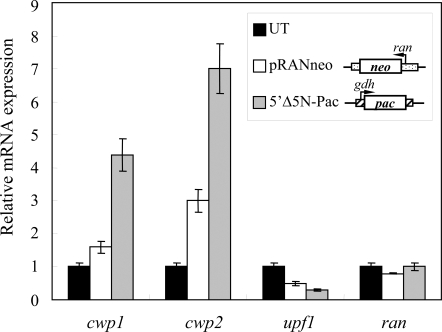
Reverse correlation between the expression levels of the *cwp1/2* and *upf1* mRNA in stable cell lines. Diagrams of the pRANneo and 5′Δ5N-Pac plasmids for stable transfection are shown in inset. The *neo* or *pac* gene (open box) is under the control of the 5′- and 3′-flanking regions of the *ran* (dotted box) or *gdh* gene (striated box). Total RNA blots made from vegetative untransfected cells (UT), pRANneo or 5′Δ5N-Pac transfectants were hybridized with specific probes as indicated. Hybridization signals were imaged and quantified as indicated in Materials and Methods. The results are expressed as relative expression level over untransfected control. Values are shown as mean±standard error.

Previously we found that the levels of the *upf1* mRNA decreased with increasing levels of the *cwp1* and *cwp2* mRNA during encystation [34]. We also demonstrated that stable transfection systems can increase the levels of the *cwp1*, *cwp2* and *cwp3* transcripts during vegetative growth [35]. We further asked whether stable transfection influenced the *upf1* gene expression. We found that the level of the *upf1* mRNA decreased by ∼50–70% (*P*<0.05) in cells stably transfected with the pRANneo or 5′Δ5N-Pac, which contain the neomycin or puromycin selective marker, relative to untransfected cells (Fig. 2). As reported previously, the levels of the *cwp1/2* mRNA increased significantly and the levels of the *ran* and ribosomal mRNA did not change significantly in the transfected cell lines (Fig. 2) [35].

### Overexpression of UPF1 reduced the levels of cyst wall protein 1 and cyst formation

**Figure 3:**
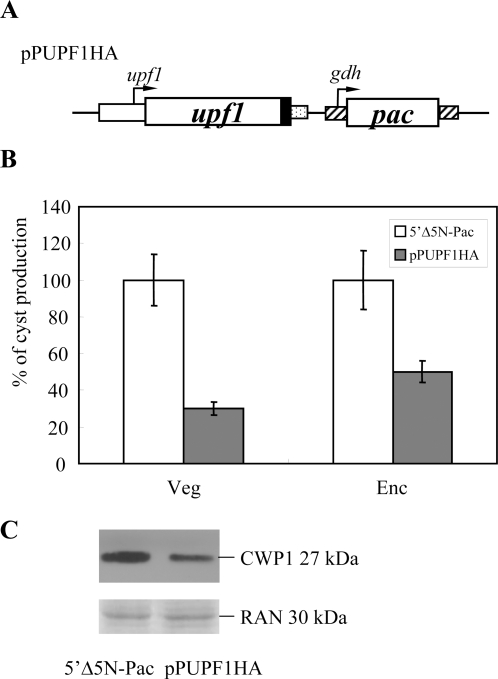
Overexpression of UPF1 reduced the levels of CWP1 and cyst formation. (A) Diagrams of the pPUPF1HA plasmid. The *pac* gene (open box) expression cassette is the same as in Fig. 2. The *upf1* gene is under the control of its own 5′-flanking region (open box) and the 3′-flanking region of the *ran* gene (dotted box). The filled black box indicates the coding sequence of the HA epitope tag. (B) Overexpression of UPF1 reduced the levels of cyst formation. The 5′Δ5N-Pac and pPUPF1HA stable transfectants were cultured in growth medium to late log/early stationary phase (Veg). Cyst count was performed on these late log/early stationary phase cultures (1.5×10^6^ cells/ml). In another study, the 5′Δ5N-Pac and pPUPF1HA stable transfectants were cultured in encystation medium for 24 h and then subjected to cyst count (Enc). The sum of total cysts is expressed as relative expression level over control. Values are shown as mean±standard error. (C) Overexpression of UPF1 reduced the CWP1 level. The 5′Δ5N-Pac and pPUPF1HA stable transfectants were cultured in encystation medium for 24 h and then subjected to SDS-PAGE and Western blot. The blot was probed by anti-CWP1 antibody. Equal amounts of proteins loaded were confirmed by detection of giardial RAN protein. Representative results are shown.

Because of the reverse correlation between the levels of the *cwp1/2* and *upf1* mRNAs as described above, we further investigated the effect of *giardial* UPF1 on cyst formation. We stably transfected the UPF1 expression plasmid pPUPF1HA into *G. lamblia* (Fig. 3A). The UPF1-HA protein was expressed in the stable transfectants as detected in immunofluorescence assays [34]. No significant change in growth rate was observed in the pPUPF1HA cell line (data not shown). We found that the cyst number in the vegetative or encysting pPUPF1HA cell line decreased by ∼30% or ∼50% (*P*<0.05) relative to the control cell line which only carries the *pac* gene (5′Δ5N-Pac) (Fig. 3B). We further asked whether the levels of cyst wall protein 1 decreased with the decrease of the cyst formation. As shown by Western blot analysis, UPF1 overexpression also resulted in a reduction of the levels of CWP1 protein (Fig. 3C). As a control, similar levels of intensity of the giardial RAN protein (∼30 kDa) were detected by anti-human RAN antibody (Fig. 3C). The results suggest that UPF1 may function in reducing the level of CWP1 and cyst formation.

### Overexpression of UPF1 decreased *cwp1-3* and *myb2* mRNA stability

**Figure 4 pone-0003609-g004:**
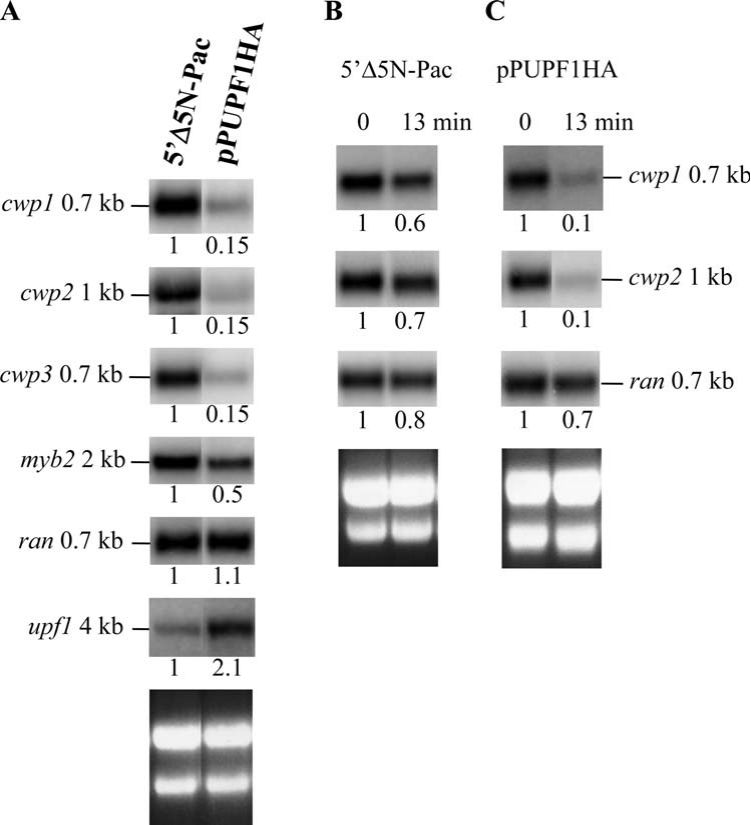
Effect of UPF1 overexpression on *cwp* gene expression. (A) Overexpression of UPF1 decreased the *cwp1-3* and *myb2* mRNA levels. Total RNA was harvested from vegetative 5′Δ5N-Pac and pPUPF1HA transfectants. Northern blots were hybridized with specific probes as indicated (upper panels). (B)(C) Overexpression of UPF1 decreased the *cwp1* and *cwp2* mRNA stability. Total RNA was harvested from either 5′Δ5N-Pac (B) or pPUPF1HA (C) transfectants during vegetative growth. The cells were treated without (0 min) or with 45 µg/ml actinomycin D for 13 min to arrest mRNA synthesis. Northern blot were hybridized with specific gene probes as indicated (upper panels). Ribosomal RNA loading controls are in the bottom panels. Representative results are shown. The numbers show the relative activity, which reflects expression relative to that in controls. The *cwp1* and *cwp2* signals from Fig. 4B and C were a long exposure to show the difference in the AcD treated and untreated samples.

We further investigated whether UPF1 overexpression can influence the expression of *cwp* and other endogenous genes. We found that the levels of native *cwp1*, *cwp2*, *cwp3*, or *myb2* mRNA in the vegetative pPUPF1HA cell line decreased by ∼85% or ∼50% (*P*<0.05) relative to the control cell line which only carries the *pac* gene (Fig. 4A). The *ran* mRNA levels did not change significantly in the pPUPF1HA cell line (Fig. 4A). The *upf1* mRNA levels increased significantly (*P*<0.05) in the pPUPF1HA cell line (Fig. 4A).

We further investigated whether the decrease of *cwp* mRNA levels in the pPUPF1HA transfectants was due to the decrease of mRNA stability. We found that treatment of the pPUPF1HA transfectants with 45 µg/ml of actinomycin D for 13 min decreased mRNA levels of the *cwp1* and *cwp2* genes by ∼90% (*P*<0.05) (Fig. 4C). As a control, treatment of the control cell line with actinomycin D for 13 min decreased mRNA levels of the *cwp1* and *cwp2* genes by 30–40% (*P*<0.05) (Fig. 4B). Therefore, the *cwp1* and *cwp2* mRNAs exhibited a half-life of <13 min when the *upf1* was overexpressed, while these mRNAs exhibited a half-life of >13 min in the control cell line. The *cwp3* and *myb2* mRNA stability also decreased significantly in the pPUPF1HA transfectants as compared with that in the control cell line (data not shown). The *ran* mRNA stability did not change significantly in the pPUPF1HA transfectants as compared with that in the control cell line (Fig. 4C). The results indicate that UPF1 can decrease *cwp1-3* and *myb2* mRNA stability.

### Overexpression of UPF1 changed transcript levels of other endogenous genes

**Figure 5 pone-0003609-g005:**
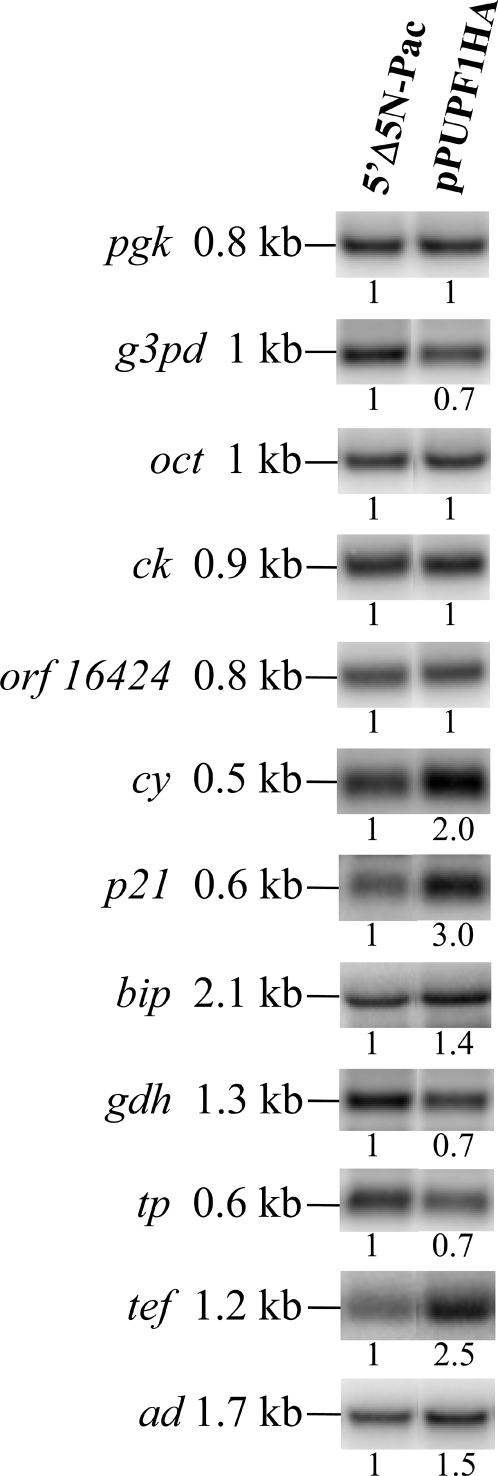
Effect of UPF1 overexpression on endogenous gene expression. Total RNA blots made from the 5′Δ5N-Pac and pPUPF1HA transfectants were hybridized with specific gene probes as described. Equal RNA loading was confirmed by ethidium bromide staining of ribosomal RNA (data not shown). Representative results are shown. The numbers show the relative activity, which reflects expression relative to that in controls.

In the previous studies, we have found that the expression of the *cwp1*, *cwp2*, *myb2* genes was upregulated in stable cell line with drug selection and that the expression of other eight genes was also upregulated [35]. They encodes enzymes involved in anaerobic glycolysis, phosphoglycerate kinase (PGK) and glyceraldehyde-3-phosphate dehydrogenase (G3PD), enzymes for arginine hydrolysis, ornithine carbamoyltransferase (OCT) and carbamate kinase (CK), enzymes involved in protein folding, cyclophilin (CY), co-chaperone-like protein p21, and Bip, and open reading frame (ORF) 16424 with unknown function [1], [35], [36]. We wished to understand the importance of UPF1 for expression of these genes. To achieve this goal, we compared expression of these genes in the UPF1 overexpressed cell line and the control cell line. Of these eight genes, three were upregulated by UPF1 overexpression, including *cy*, *p21*, and *bip* (*P*<0.05) (Fig. 5). Interestingly, one gene was downregulated by UPF1 overexpression (*g3pd*, *P*<0.05) (Fig. 5). The transcript levels of the other four genes, *pgk*, *oct*, *ck*, and *orf 16424*, were not changed significantly by UPF1 overexpression (Fig. 5). We also found that two other genes with no change in mRNA levels in the stable cell lines, glutamate dehydrogenase (*gdh*), and thioredoxin peroxidase (*tp*), were downregulated by UPF1 overexpression (*P*<0.05) (Fig. 5). In addition, we found two newly identified genes, *translation elongation factor* (*tef*, γ subunit of translation elongation factor 1B) [37] and *arginine deiminase* (*ad*) [1], were upregulated by UPF1 overexpression (*P*<0.05) (Fig. 5).

### Overexpression of UPF1 decreased mRNA levels of vector-expressed *cwp1* gene

**Figure 6 pone-0003609-g006:**
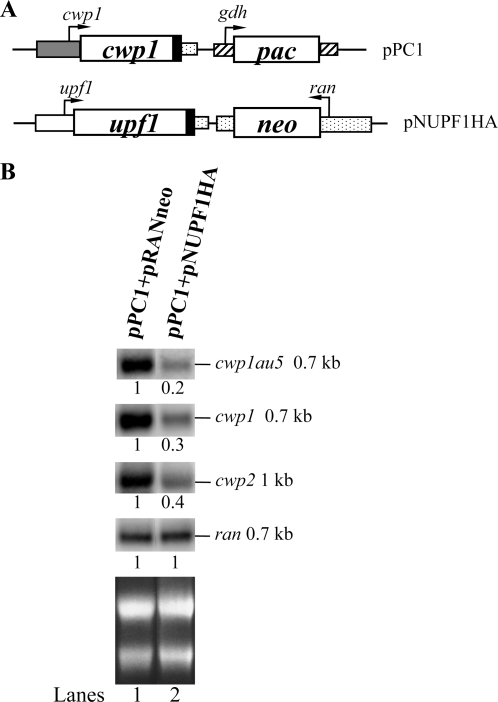
Overexpression of UPF1 decreased mRNA levels of vector-based *cwp1* gene. (A) Diagrams of the pPC1 and pNUPF1HA plasmids. The *neo* or *pac* gene (open box) expression cassette is the same as in Fig. 2. In pPC1, the *cwp1* gene (open boxes) is flanked by its own 5′-flanking region (gray box) and 3′-flanking region of the *ran* gene (dotted box) and the filled black box indicates the coding sequence of the AU5 epitope tag. In pNUPF1HA, the *upf1* gene is under the control of its own promoter (open box) and the 3′-flanking of the *ran* gene (dotted box) and the filled black box indicates the coding sequence of the HA epitope tag. (B) Northern blot analysis of au5 tagged *cwp1* transcripts in vegetative cells (upper panel). Total RNA blots made from pPC1+pRANneo or pPC1+pNUPF1HA transfectants were hybridized with the au5 specific probe (au5hyb), and specific gene probes as indicated (upper panels). Ribosomal RNA loading controls are in the bottom panel. Representative results are shown. The numbers show the relative activity, which reflects expression relative to that in controls.

We next tested whether overexpression of UPF1 influenced vector-expressed *cwp1* gene. We stably transfected the UPF1 expression plasmid pNUPF1HA (Fig. 6A) together with construct pPC1 in which the *cwp1* gene is controlled by its own promoter and contains an AU5 epitope tag at their C terminus (Fig. 6A). The CWP1-AU5 protein was expressed in the stable transfectants as detected in immunofluorescence assays and Western blot analysis (data not shown). Northern blot analysis showed that the levels of the *cwp1-au5* mRNA decreased by ∼80% (*P*<0.05) in the pPC1+pNUPF1HA co-transfectants relative to the pPC1+pRANneo control cell line during vegetative growth (Fig. 6B). The levels of the *cwp1* (including endogenous *cwp1* and vector-derived *cwp1-au5*) and *cwp2* mRNA also decreased by ∼60–70% (*P*<0.05) in the UPF1 overexpressed cell line, pPC1+pNUPF1HA (Fig. 6B). The results indicate that overexpression of UPF1 not only can decrease the expression of the endogenous *cwp* genes but also can decrease the expression of the vector-expressed *cwp1* gene.

### Mapping of the region in the *cwp1* gene needed for the UPF1 dependent decay

**Figure 7:**
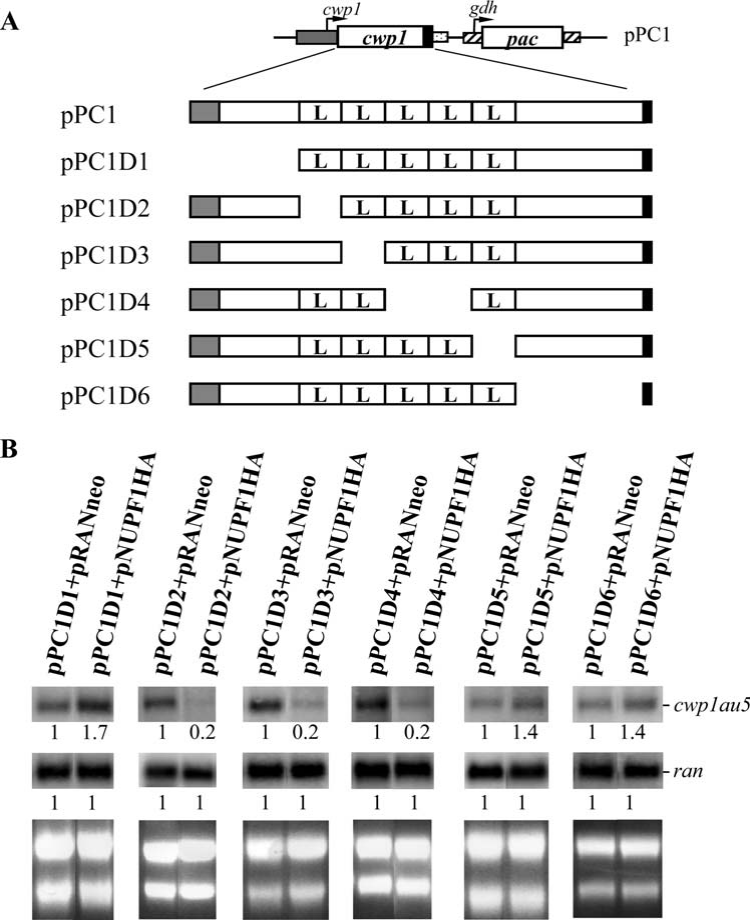
Mapping of the region in the *cwp1* gene needed for the UPF1 dependent decay. (A) Diagrams of the pPC1 and plasmids for CWP1 deletion mapping. The *pac* or *cwp1* gene (open box) expression cassette is the same as in Fig. 2 and Fig. 6, respectively. The predicted signal peptide is in gray. The LRRs are indicated as open boxes labeled “L”. (B). Analysis of au5 tagged *cwp1* transcripts in vegetative cells. The pPC1D1-6+pRANneo and pPC1D1-6+pNUPF1HA transfectants were cultured in encystation medium for 24 h and then subjected to Northern blot analysis. Total RNA blots were hybridized with the au5 specific probe (au5hyb), and *ran* gene probe (upper panels). Ribosomal RNA loading controls are in the bottom panel. Representative results are shown. The numbers show the relative activity, which reflects expression relative to that in controls.

We further determined the region within the *cwp1* mRNA responsible for the UPF1 dependent decay by constructing a series of deletions (Fig. 7A). Deletion of the sequence encoding the first, second or third to fourth LRRs (nucleotides 193–264, construct D2; nucleotides 265–345, construct D3; nucleotides 346–474, construct D4, Fig. 7A) still resulted in a significant decrease of *cwp1-au5* mRNA in the pNUPF1HA co-transfectants relative to the control cell line (Fig. 7B). However, deletion of the sequence encoding the region N terminal to the LRRs (nucleotides 4–192, construct D1, Fig. 7A), the fifth LRR (nucleotides 475–552, construct D5, Fig. 7A) or the region C terminal to the LRRs (nucleotides 553–723, construct D6, Fig. 7A) resulted in an increase of the levels of the *cwp1-au5* mRNA to ∼1.4 or 1.7-fold (*P*<0.05) in the pNUPF1HA co-transfectants relative to the control cell line (Fig. 7B). The results indicate that 5′ (nucleotides 4–192) or 3′ (nucleotides 475–723) sequence of the *cwp1* gene may contain the sequence responsible for the UPF1 dependent decay.

## Discussion

In addition to RNA surveillance, NMD factors also function in regulating the abundance of some naturally occurring mRNAs [23], [28], [38]. Hundreds or thousands of wild-type NMD targets that may be up-regulated or down-regulated by NMD factors have been identified in yeast and humans [28], [29], [39]. Their changes in mRNA abundance may be correlated (direct targets of NMD) or not correlated (indirect targets of NMD) with changes in the mRNA stability [28], [39]. For example, abundance of one subset of wild type mRNAs increased in *upf1* null mutant, including PPR1, URA1, URA3, and URA4 mRNAs [28], [40]. However, only PPR1 is the direct target of NMD because only PPR1 mRNA stability increased in *upf1* null mutant [28], [40]. PPR1 is a positive transcriptional activator for these URA genes. Altered half-life of this regulatory protein could indirectly influence the abundance of the mRNAs of the downstream targets (URA genes) [28], [40]. An UPF1 dependent destabilizing element (UDE) was mapped to a region located within the 5′-untranslated region and the first 92 bases of the PPR1 coding region [40]. Similarly, one of the UDEs was mapped to a region located within the 5′ (nucleotides 4–192) sequence of the *cwp1* gene.

In *G. lamblia*, we identified the four encystation-induced genes, *cwp1*, *cwp2*, *cwp3*, and *myb2*, as wild-type targets of NMD. These four genes may be the direct targets of NMD because overexpression of UPF1 led to decreased levels of their mRNA stability. In addition, UDEs were mapped to a region located within the 5′ (nucleotides 4–192) or 3′ (nucleotides 475–723) sequence of the *cwp1* gene. It is possible that the *cwp2*, *cwp3* and *myb2* genes also have UDEs and that their mRNAs are targeted by UPF1-dependent decay. UPF1 may coordinately down-regulate the encystation-induced *cwp1-3* and *myb2* genes during vegetative growth. Because Myb2 acts as a positive transcriptional activator for these *cwp* genes [8], a decrease of the level of the *myb2* transcripts that may lead to a decrease of the levels of the Myb2 protein and then results in a decrease of the *cwp* transcripts. In addition, we have shown that the levels of UPF1 decreased significantly during encystation [34]. During encystation, the up-regulation of these encystation-induced genes may be correlated with the presence of the lower level of UPF1 protein. Interestingly, our results show that the levels of the *cwp1* and *cwp2* mRNAs were lower in the cells containing luciferase nonsense transcripts. This could be due to the down-regulation of the *cwp* genes by the presence of the higher level of UPF1 protein upon NMD induction.

It is interesting to know how NMD factors recognize their targets. In yeast, it has been thought that NMD promotes rapid decay of the nonsense-containing mRNA through interaction of a RNA-binding protein(s) with specific RNA elements [12]. Heterogeneous nuclear ribonucleoprotein 1 (HRP1) may be a marker protein that binds to the downstream element of the nonsense mutation and interacts with NMD factors [12]. On the other hand, wild-type mRNAs without premature stop codon were also regulated by a UPF1-dependent mechanism [38], [41]. Four natural targets for an RNA-binding protein, Stau1, were identified in humans, including ADP ribosylation factor 1, c-JUN, SERPINE1, IL7R, and GAP43 mRNAs. Stau1 can bind to the 3′-untranslated region of these targets' mRNAs for Stau1-mediated mRNA decay, which depends on translation and recruitment of the NMD factor UPF1 [38], [41]. Short untranslated regions are typical of giardial transcripts [3]. Interestingly, one of the UDEs was mapped to a region located within the 3′ (nucleotides 475–723) sequence of the *cwp1* gene. It is possible that *G. lamblia* may have similar RNA binding proteins as marker proteins for abnormal or natural NMD targets. Blast searches of the *G. lamblia* genome databases identified a match for HRP1, which has five RNA binding domains [34]. However, it is very different from the yeast HRP1, which has two RNA binding domains. Further studies will be required to identify the RNA binding proteins and elucidate their roles in NMD.

In addition to encystation-induced genes, we also found that UPF1 may be involved in regulating transcripts of many different genes. Overexpression of UPF1 led to enhanced levels of five of twelve genes, including *cy*, *p21*, *bip*, *tef* and *ad*. Three genes were downregulated, including *g3pd*, *gdh*, and *tp*. The other four genes tested were not changed, including *pgk*, *oct*, *ck*, and *orf 16424*. The affected genes encode proteins involved in protein translation (TEF) [37], protein folding (CY, p21, and Bip) [35], cell metabolism (AD, G3PD, GDH, and TP) [1], [36]. They may be direct or indirect targets of NMD and this requires further investigation.

NMD may increase translation accuracy because NMD factors including UPF1-3 could enhance translation termination at a nonsense codon through interaction with the termination release factors in yeast [20], [24]–[26]. NMD requires translation since its mechanism depends on the recognition of the nonsense mutations by the translational machinery [13]. NMD factors including UPF1-3 also function in stimulating translation in human [42]. In addition, NMD factors including UPF1-3 may increase translation through down-regulation of Ebs1p, which is a global inhibitor of translation in yeast [43]. This occurs without a change in the *EBS1* mRNA stability, indicating that *EBS1* is an indirect target of NMD and it may be targeted by NMD-regulated transcription factors. In this study, we also found that UPF1 may be involved in upregulating transcripts of *translation elongation factor* (*tef*, γ subunit of translation elongation factor 1B) [37]. This suggests that the giardial NMD factors may function in enhancing translation through upregulation of *tef*. Therefore, we provided a new example of an NMD target whose product increases translation initiation which is required for NMD.

Our results indicate that NMD can affect some endogenous genes involved in differentiation, metabolism, and protein translation and folding in the early-diverging protozoan *G. lamblia*. Our findings also lead to greater understanding of the regulation of mRNA stability of the genes involved in cyst wall pathway and provide a model to investigate the mechanism of cell differentiation.

## Materials and Methods

### *G. lamblia* culture

Trophozoites of *G. lamblia* WB (ATCC 30957) clone C6 were cultured in modified TYI-S33 medium [44] and encysted as previously described [7]. Cyst count was performed on vegetative cultures as previously described [35]. Cyst count was also performed on 24 h encysting cultures. In experiments exposing *G. lamblia* vegetative trophozoites to actinomycin D, trophozoites were cultured in medium 45 µg/ml actinomycin D (in PBS) for indicated time in the legends of Figures 4B and C.

### RNA extraction and Northern blot analysis

Total RNA was extracted from *G. lamblia* clones C6 at the indicated differentiation stages in the legends of Figures 1, and 3–7 using TRIzol reagent (Invitrogen). For Northern blot analysis, 10 µg total RNA was fractionated and transferred to charged Nylon membranes (Biodyne B membrane, Pall). Full-length coding region probes of luciferase, *cwp1* (GenBank accession no. **U09330**), *cwp2* (GenBank accession no. **U28965**), *cwp3* (GenBank accession no. **AY061927**), *myb2* (GenBank accession no. **AY082882**), *ran* (GenBank accession no. **U02589**), *upf1* (GenBank accession no. **DQ861427**), *phosphoglycerate kinase* (*pgk*, GenBank accession no. for genomic DNA **XM_762975**), *glyceraldehyde-3-phosphate dehydrogenase* (*g3pd*, GenBank accession no. for genomic DNA **M88062**), *ornithine carbamoyltransferase* (*oct*, GenBank accession no. for genomic DNA **XM_765341**), *carbamate kinase* (*ck*, GenBank accession no. for genomic DNA **XM_765099**), *orf 16424* (GenBank accession no. for genomic DNA **XM_764168**; *orf 16424* in *G. lamblia* genome database, http://www.giardiadb.org/giardiadb/)(Morrison et al., 2007), *cyclophilin* (*cy*, GenBank accession no. for genomic DNA **XM_774688**), *p21* (GenBank accession no. for genomic DNA **XM_762782**, for protein **XP_767875**), *bip* (GenBank accession no. for genomic DNA **XM_766560**, for protein **XP_771653**), *glutamate dehydrogenase* (*gdh*, GenBank accession no. for genomic DNA **XM_773614**), *thioredoxin peroxidase* (*tp*, GenBank accession no. for genomic DNA **XM_774576**)[36], γ subunit of translation elongation factor 1B (*tef*, **GenBank accession no. for genomic DNA XP_778603**), and *arginine deiminase* (*ad*, **GenBank accession no. for genomic DNA U49236**) genes were prepared by PCR amplification of genomic DNA using primers lucF (ATGGAAGACGCCAAAAAC) and lucR (TTACACGGCGATCTTTCC), cwp1F (ATGATGCTCGCTCTCCTT) and cwp1R (TCAAGGCGGGGTGAGGCA), cwp2F (ATGATCGCAGCCCTTGTT) and cwp2R (TCACCTTCTGCGGACAAT), cwp3F (ATGTTTTCTCTGCTTCTT) and cwp3R (TTATCTGTAGTAGGGCGG), myb2F (ATGTTACCGGTACCTTCT) and myb2R (TCAGGGTAGCTTCTCACG), ranF (ATGTCTGACCCAATCAGC) and ranR (TCAATCATCGTCGGGAAG), upf1F (ATGGAGCCTTGTGCATTG) and upf1R (CTATGCCTTAGGAATTAC), pgkF (ATGTCCTTAGCGAAGCTCTCC) and pgkR (CTTCTTGTCAGACAGTCTGAT), g3pdF (ATGCCTATTCGCCTCGGAATC) and g3pdR (GCAGCCCTTGGACCCGACGTA), octF (ATGCCGTTCAAGCAGACCCGC) and octR (CTCCATCTTGCAGTCATGCAA), ckF (ATGTCGGCAGGGAAAACGGTT) and ckR (ATCCTTGATGATGCGGGTCCC), 16424F (CACCATGAGTAGAACGCCAAAC) and 16424R (GTAGCGACGATTACCGGA), cyF (ATGAACTCTCCAGTTTCTGAC) and cyR (CTGGAGCACGCCACAGTCGGC), p21F (ATGCACCATCCGACGATCTA) and p21R (CTCCTCTGCCTTCTCTTCGCC), bipF (ATGACGTCTAGTCACGTTAA) and bipR (GAGTTCATCTTTTTCTGCAT), gdhF (ATGCCTGCCCAGACGATCGAG) and gdhR (CACGCAGCCCTGCTCGATCAT), tpF (ATGCCCGTCCCCATCCCCGGC) and tpR (CTTCTTGAACGTCTTGGAGAA), tefF (ATGCAGATCACAGGCAGTCAG) and tefR (CTAGTGCCAAGTCTCCCCATC), adF (ATGACTGACTTCTCCAAGGAT) and adR (TCACTTGATATCGACGCAGAT), respectively. Radiolabeled probes were prepared using the Rediprime II kit (GE Healthcare). An oligonucleotide probe complementary to the AU5 tag coding sequence and its flanking region (au5hyb, GAATTCTCACTTGAGGTAGAAATCGGTAGGCGGGGTGAGG, AU5 tag coding sequence is underlined) was end-labeled using [γ-^32^P] ATP and T4 polynucleotide kinase. The membranes were hybridized and washed as previously described [45]. Equal loading was confirmed by reprobing the Northern blots with radiolabeled ribosomal DNA. The ribosomal DNA fragment for large subunit ribosomal RNA (**X05397**) was amplified by PCR using primers RIBOF (GGCCTGCCCCTCGCCCGC) and RIBOR (CCCCTCAGTCCTCCGGGG) and a genomic DNA template. Radiolabeled ribosomal DNA probes were prepared as described above. For detection, the blots were exposed to a storage phosphor screen and the radioactive signals were quantitated using a Typhoon Trio™ Variable Mode Imager (GE Healthcare). Two independently generated stably transfected lines were made from each construct and each of these cell lines was assayed three separate times. The results are expressed as relative expression level over control. Student's *t*-tests were used to determine statistical significance of differences between samples.

### Plasmid construction

All constructs were verified by DNA sequencing with a BigDye Terminator 3.1 DNA Sequencing kit and an ABI 3100 DNA Analyser (Applied Biosystems). Plasmid 5′Δ5N-Pac was a gift from Dr. Steven Singer and Dr. Theodore Nash [46]. Plasmids pRANneo, pPW1, pPW1m, pPC1, pPUPF1HA, and pNUPF1HA have been described previously [7], [34], [35], [47]. A *Nhe*I/*Cla*I fragment containing the *luciferase* gene, 32-bp *ran* promoter and two copies of a 19-bp *tet* operator sequence from pPop2N [34] was replaced by the *Nhe*I/*Cla*I excised *luciferase* gene and *α2-tubulin* promoter from pNT5, resulting in pPT5. An *Nco*I/*Cla*I fragment containing the wild type *luciferase* gene from pPT5 [34] was replaced by the *Nco*I/*Cla*I excised mutated *luciferase* gene from pPW1m, resulting in pPT5m. For constructing pPC1D1, a PCR with oligonucleotide cwp1D1NF (GGCGCCATGGATGCCCTGGATCTTTCGGACATG) and ran3C (GCGGATCGATGTAACGAACCGCTAGAAG) generated a 0.8-kb product that was digested with *Nco*I and *Cla*I. Another PCR with primers T3 (ATTAACCCTCACTAAAG) and cwp1D1NR (GGCGCCATGGCCCTGATATTTTATTTCTGTG) generated a 0.3-kb PCR product that was digested with *Nco*I and *Nhe*I and cloned into *Nhe*I/*Cla*I digested pPop2N with the 0.8-kb *Nco*I/*Cla*I fragment. The resulting pPC1D1 contains a *cwp1* gene lacking the coding sequence for the predicted signal peptide sequence and the sequence N terminal to LRRs (nucleotides 4–192). For constructing pPC1D2, a PCR with oligonucleotide cwp1D2BF (GGCGGGATCCACCCTTTACTTGAGCAACAAC) and ran3C generated a 0.6-kb product that was digested with *Bam*HI and *Cla*I. Another PCR with primers T3 and cwp1D2BR (GGCGGGATCCGATAACGTAGTTATTCGAGGC) generated a 0.4-kb PCR product that was digested with *Bam*HI and *Nhe*I and cloned into *Nhe*I/*Cla*I digested pPop2N with the 0.6-kb *Bam*HI/*Cla*I fragment. The resulting pPC1D2 contains a *cwp1* gene lacking the coding sequence for the first LRR (nucleotides 193–264). Similar strategy was used to constructs pPC1D3, pPC1D4, pPC1D5, and pPC1D6, which contain *cwp1* gene with deletion of the coding sequence for the second LRR (nucleotides 265–345), third to fourth LRR (nucleotides 346–474), fifth LRR (nucleotides 475–552), and the sequence C terminal to LRRs (nucleotides 553–723), respectively.

### Transfection, luciferase assay, and Western blot analysis

Cells transfected with pN series plasmids were selected with G418 as described [47]. Stable transfectants were maintained at 150 µg/ml G418. Cells transfected with pP series plasmids containing the *pac* gene were selected and maintained with 54 µg/ml puromycin. For co-transfection assays (see Figs. 6 and 7), *G. lamblia* cells were first transfected with pP series plasmids and selected in 54 µg/ml puromycin. The stable transfectants were transfected with pN series plasmids and then the cells were doubly selected in both 150 µg/ml G418 and 54 µg/ml puromycin. After stable transfection with specific constructs, luciferase activity was determined in vegetative cells at late log/stationary phase (1.5×10^6^ cells/ml) or in 24 h encysting cells as described [45] and was measured with an Optocomp I luminometer (MGM Instruments). Two independently generated stably transfected lines were made from each construct and each of these lines was assayed three separate times. Western blots were probed with anti-CWP1 antibody (1/10,000) [48] or anti-human RAN antibody (1/5,000) (Santa Cruz Biotechnology), and detected with peroxidase-conjugated goat anti-mouse IgG (Pierce, 1/5,000) and enhanced chemiluminescence (GE Healthcare).
